# Further assessment of structural and social determinant of health effects on breast cancer care timelines: a qualitative study

**DOI:** 10.3389/fonc.2026.1887906

**Published:** 2026-08-19

**Authors:** Danielle Tomer, Sara Friedman, Risha Sheni, Dana Watnick, Della Makower, Anjuli M. Gupta, Sheldon Feldman, Maureen McEvoy, Vikas Mehta

**Affiliations:** 1 Albert Einstein College of Medicine, Bronx, NY, United States; 2 Department of Otorhinolaryngology–Head and Neck Surgery, Montefiore Medical Center/Albert Einstein College of Medicine, Bronx, NY, United States; 3 Department of Pediatrics, Albert Einstein College of Medicine, Bronx, NY, United States; 4 Department of Oncology, Montefiore Medical Center, Bronx, NY, United States; 5 Department of Surgery, Breast Surgery Division, Montefiore Medical Center, Bronx, NY, United States

**Keywords:** breast cancer, social determinants of health, social needs, time to treatment, treatment delay

## Abstract

**Background:**

Time to treatment initiation (TTI) delays result in worse cancer outcomes. Patients in under-resourced communities may experience TTI delays due to structural challenges and social determinants of health (SDOH), impacting access to timely care. We conducted a qualitative study in breast cancer patients with and without delayed TTI from an under-served community to explore barriers and facilitators to TTI from the patient perspective.

**Methods:**

We interviewed 30 patients who received curative intent breast cancer treatment at Montefiore Medical Center from 2019 to 2024. Half of the patients had delayed TTI (≥45 days), while the other half were not delayed. Interviews addressed barriers and facilitators of timely TTI, perceived timeliness of care, emotional well-being, and perceptions of the healthcare team and communication. Interviews were coded based on recurring ideas and patterns, and framework analysis was used to finalize themes.

**Results:**

Our cohort (n=30) had a mean age of 60.9 years, all whom identified as female. Most participants identified as Black or African-American (56.7%), and 16.7% identified as Hispanic/Latino. Age, race, ethnicity, tumor stage, and first treatment modality were similar between delayed and non-delayed TTI groups, whereas the distribution of pathologic diagnoses approached borderline significance. The mean TTI in delayed participants was 55.1 days and 32.7 days in the non-delayed group. Three main themes were identified: (1) Facilitators and Barriers, (2) Coping in the Interim: Waiting for Treatment Initiation, and (3) Patient Perceptions of Timeliness. Delayed patients faced financial burdens, transportation issues, and childcare challenges, hindering ability to adhere to schedules. Approximately half of the full cohort (delayed and non-delayed patients) wished their treatment began sooner, while half of the delayed TTI group expressed preference for longer TTI that afforded them time to prepare for treatment, make important decisions and seek opinions. Frequently participants found significant emotional and logistical support through family, social networks, and religion as well.

**Conclusions:**

Our study revealed that delayed patients faced financial and logistical barriers to initiating treatment. Findings from our work highlight the need for healthcare systems to better equip and engage patients in the care timeline to avoid delayed TTI.

## Introduction

Delays in time to treatment initiation for new cancer diagnoses are associated with an increased risk of mortality and recurrence (1). In an observational study across multiple cancer types, each one-week delay in treatment initiation was associated with a 1.2%-3.2% increased risk of death across breast, lung, pancreas, colorectal and renal cancers in multivariable analyses (2). A meta-analysis similarly found that a four-week delay in cancer treatment was associated with increased mortality across surgical, chemotherapeutic, and radiotherapeutic interventions for many different solid organ malignancies (3). A randomized clinical trial in patients with esophageal cancer provided supportive causal evidence for these findings, demonstrating worse survival outcomes among patients assigned to delayed treatment (10–12 weeks) compared with standard treatment (4–6 weeks) (4).

TTI in breast cancer patients is one of the most delayed among cancer types (2). The association between TTI and survival has been demonstrated in multiple observational studies of patients with breast cancer. In breast cancer patients with early-stage disease, a decline in overall survival is seen when time to surgery increases (5). Similarly, delays of greater than 3 months in time between lumpectomy and initiation of adjuvant radiation therapy are associated with higher overall and cancer-specific mortality (6). Understanding the drivers of these delays is critical to improving cancer outcomes.

Delays in time to treatment initiation disproportionately occur in under-resourced communities due to greater structural challenges and unmet needs (7, 8). A systematic review of studies across 14 countries reported that low socioeconomic status and place of residence were frequent barriers to time to treatment initiation, while initial contact with a private health facility or specialist reduced time to diagnosis (9). In an observational study of 715,210 breast cancer patients in the National Cancer Database (NCDB), Black and Hispanic patients had increased times to first treatment initiation compared with White and non-Hispanic patients (7 and 11 days longer, respectively), and uninsured patients also experienced significantly longer time intervals to surgery and radiation treatments compared with privately insured patients (23 days and 36 days longer, respectively) (10). Another study of breast cancer patients undergoing neoadjuvant chemotherapy found that each additional hospital visit increased time to treatment initiation by 14% (11), highlighting the need for coordination of care and multidisciplinary clinics to reduce visits and delays in treatment initiation.

Furthermore, psychosocial stress related to structural barriers or coping with a new cancer diagnosis may influence TTI. Financial toxicity has been associated with delays in cancer treatment and care, as well as maladaptive coping mechanisms that worsen outcomes (12). Fear is another common emotion-driven barrier to initiating cancer care (13). In a qualitative study of breast cancer patients, emotional despair and unfulfilled emotional, financial, logistical and spiritual support was observed in patients with delayed chemotherapy initiation (14). Understanding the psychosocial factors that shape patients’ experiences is essential to identifying factors that contribute to delayed TTI.

To better understand the patient and system-level factors that impact delivery of oncologic care, it is critical to understand the reasons for delays from the patient’s perspective. As has been demonstrated in a large study by Fiori et. al., patients who receive care at our institution often face challenges with social determinants of health, which impact their ability to engage with the healthcare system (15). Studies within our oncologic population have also demonstrated that delays in cancer TTI are associated with unmet social needs, lower median household income, and greater medical morbidities (8, 16). Our aim was to better understand how patient, social, and system-level factors influence patient care trajectories and differ between patients with and without delayed TTI. We also sought to better understand and characterize the patients’ experience, mindset and understanding of their cancer diagnosis and journey during this pivotal and impactful TTI period. To explore this, our study conducted qualitative narrative interviews with breast cancer patients about their cancer care treatment trajectories to further understand barriers and facilitators to treatment initiation that may not be identifiable through simple electronic health record data capture.

## Materials and methods

### Participants and procedures

This was a qualitative, prospective IRB approved study that was conducted on breast cancer patients who were diagnosed and treated at Montefiore Medical Center (MMC), an academic medical center serving primarily a population that resides in the Bronx, with almost one-third of residents living in poverty, and the county that is routinely cited as the least healthy in the state (17, 18). We obtained data on basic demographic, initial treatment modality, date of histopathologic diagnosis, and date of treatment initiation from patients’ electronic medical records. We defined early-stage disease as stages 0–2 and late stage as stages 3–4 using clinical or pathologic prognostic stages based on AJCC’s 8^th^ edition staging system. TTI was calculated as the difference between first treatment date and histopathologic diagnosis date. Treatment date was defined as the date of the first surgery, first chemotherapy infusion treatment, or first endocrine therapy treatment. Delayed treatment initiation was defined as >45 days to treatment initiation, which was determined based on previous studies related to TTI in our patient population and other populations (16, 19).

Patients met our inclusion criteria if they had a diagnosis of first-time breast cancer and received care at MMC from 2019-2024. Patients were excluded if this was not their first cancer diagnosis or if they were followed in high-risk outpatient breast cancer clinic already due to suspicious breast findings in the past, a strong family history of breast cancer, or a previously identified genetic mutation that increases cancer risk (e.g. BRCA mutations). Patients were also excluded if treatment was not done with curable intent (i.e. palliative) and if the diagnostic biopsy was not done at MMC. We purposively sampled a total of 30 participants: half (n=15) had timely TTI and half (n=15) had delayed TTI, defined as >45 days to treatment initiation. The sample size was determined *a priori* to allow balanced group comparison, with 15 participants per group considered sufficient to achieve thematic saturation, based on prior qualitative literature suggesting that saturation is typically reached within 9–17 interviews (20).

### Interviews and data collection

Participants who met our inclusion criteria were recruited to our study at the Montefiore Einstein Cancer Center when attending an appointment with their surgical or medical breast oncology provider between 2023-2024. At the end of their clinic visit, their physician informed them about our study researching delays in breast cancer treatment and asked patients if they were interested in participating. Patients who expressed interest were informed of the purpose of the study and the interviewer’s role. Prior to conducting interviews, we obtained written informed consent and reassured participants that their responses would remain confidential and that participation would not affect their care. No relationship was established between the interviewers and participants prior to the study, and interviewers were not involved in their medical care. Interviewer characteristics reported were limited to gender, professional role, and training. The number of participants who declined participation was not documented.

Interviews took place in a private room following their clinic appointment, with no individuals other than the participant and interviewer(s) present. Interviews were conducted by two female medical students (RS and DT), who were trained in qualitative research methods and semi-structured interviewing techniques. RS started independently conducting interviews first, and two joint interviews were conducted by both interviewers before DT conducted interviews independently, to ensure consistency in interviewer technique. In total, each interviewer conducted 14 interviews independently. Interviews were conducted in a semi-structured format using a topic guide developed by the authors to address barriers and facilitators of timely treatment initiation (**Supplementary Material 1**) and interviewers were trained to follow-up on emergent topics related to TTI. All interviews were conducted in English. Included topics explored factors such as transportation access, finances, and convenience of scheduling appointments. Interviews also addressed patients’ perceived timeliness of their care and preferences for earlier or later treatment. Other related topics explored included emotional well-being while waiting for treatment initiation, perceptions of the healthcare team and the communication they received. Interview duration varied from 20 min to 1 hour, reflecting differences in the level of detail participants provided in response to the interview questions. Interviews were audio-recorded for later transcription. Field notes were not made during the interviews, as verbatim transcript data was sufficient to address the study’s aims. The transcripts were not returned to participants for review due to feasibility constraints. Participants were each given a $50 gift card for their participation. No repeat interviews were conducted. No participants dropped out of the study.

### Data management and analysis

We conducted framework analysis, a research method used to effectively summarize qualitative data, identify patterns, and generate descriptive conclusions and key themes (21). Our framework analysis team consisted of the first four authors (DT, SF, RS, DW). An experienced qualitative methodologist (DW), who did not participate in data collection, interviews, or coding, provided methodologic oversight and guidance for the framework analysis and theme development process to enhance quality assurance and analytic depth. Analysis process took place in 4 stages: transcription, familiarization, coding framework, and final application (21).

For stage one, audio recordings were transcribed into text using open-source Whisper model, run locally on a GPU (22). All 30 transcripts were manually reviewed and compared verbatim against the original audio recordings, with corrections made to ensure complete accuracy. For familiarization, each transcript was analyzed by a single member of the research team, who completed a case memo. Case memos included defining features of the interview and a summary of the main themes, focusing on four key domains: barriers and facilitators of treatment initiation, perceived timeliness of care, emotional well-being from diagnosis to treatment, and perceptions of healthcare team/system. Each case memo included a reflexivity note that detailed interviewers’ personal experiences that may have shaped data collection and interpretation. Interviews were conducted by medical students whose educational and social backgrounds differed from those of the patients they interviewed. In addition, the students did not have clinical experience directly providing breast oncology care, which may have influenced data collection and interpretation. After case memo completion, the transcripts and corresponding memos were reviewed and discussed during group meetings by the analysis team to collaboratively identify initial “repeating ideas” (23). Code saturation appeared prior to completion of all 30 transcripts, as no new repeating ideas or codes were identified in later transcripts. Repeating ideas were further distilled into a preliminary codebook, including parent and child codes and associated definitions. For the coding phase, a final codebook was then uploaded into Dedoose (Version 10.0.35, Los Angeles, CA), a web-based qualitative data analysis tool, where all transcripts were re-analyzed and indexed based on the agreed-upon codebook. Inter-coder reliability was not calculated, as the “repeating ideas” and codebook were developed through iterative, collaborative discussion, and the coding process was designed to incorporate multiple coder perspectives rather than to achieve coder agreement. Finally, for the final application stage, coded excerpts were exported and charted into a framework matrix. Excerpts were mapped, interpreted and iteratively reviewed, in order to refine final themes and sub-themes derived from the initial repeating ideas and codebook. Following analysis of the full sample, data was stratified into separate matrices for delayed and non-delayed participants to allow systematic comparison of patterns within and across groups and to identify similarities and differences in themes. Final themes were reviewed by the analytic team to ensure they accurately reflected coded data. The findings from this study were reported in accordance with the Consolidated Criteria for Reporting Qualitative Research (COREQ) 32-item checklist (24).

## Results

### Patient characteristics

Our cohort had a total of 30 patients with a mean age (standard deviation) of 60.9 (10.9) years. Our population was 100% female with 56.7% of the cohort identifying as Black or African-American and 16.7% identifying as Hispanic/Latino; additional race/ethnicity distributions are presented in **Table 1**. The initial treatment modality was lumpectomy in 50% of the cohort, neo-adjuvant chemotherapy in 43.3%, and mastectomy in 6.7%. Most patients presented with invasive ductal carcinoma (83.3%) at an early-stage (83.3%). The mean time to treatment initiation (SD) was 43.9 (13.3) days, with an average of 55.1 (8.2) days in the delayed group and 32.7 (5.5) days in the non-delayed group. Participants in the delayed and not-delayed groups had similar distributions in age, race, ethnicity, first treatment modality, and tumor stage. There was a borderline significant difference in the distribution of pathologic diagnoses. (**Table 1**).

**Table 1:** Demographics and clinical characteristics for participants with delayed time to treatment initiation, non-delayed participants, and the total cohort.

	Delayed (N=15)	Not delayed (N=15)	Total (N=30)	p-value*
Time to Treatment Initiation, Mean(SD)	55.1 (8.2)	32.7 (5.5)	43.9 (13.3)	<0.001
Age, Mean(SD)	58 (11)	63.8 (10.3)	60.9 (10.9)	0.147
Race, n (%)				
White	4 (26.7)	1 (6.7)	5 (16.7)	
Black	8 (53.3)	9 (60)	17 (56.7)	
Other	1 (6.7)	3 (20)	4 (13.3)	
Declined	2 (13.3)	2 (13.3)	4 (13.3)	0.431
Ethnicity, n (%)				
Hispanic	1 (6.7)	4 (26.7)	5 (16.7)	
Not Hispanic	12 (80)	8 (53.3)	20 (66.7)	
Declined	2 (13.3)	3 (20)	5 (16.7)	0.329
Pathologic diagnosis, n (%)				
Ductal Carcinoma in Situ	3 (20)	0 (0)	3 (10)	
Invasive Ductal Carcinoma	10 (66.7)	15 (100)	25 (83.3)	
Invasive Lobular Carcinoma	1 (6.7)	0 (0)	1 (3.3)	
Invasive Mammary Cancer	1 (6.7)	0 (0)	1 (3.3)	0.042
Tumor stage, n (%)				
Early	13 (86.7)	12 (80)	25 (83.3)	
Late	2 (13.3)	3 (20)	5 (16.7)	1.0
First treatment modality, n (%)				
Lumpectomy	8 (53.3)	7 (46.7)	15 (50)	
Mastectomy	2 (13.3)	0 (0)	2 (6.7)	
Neoadjuvant Chemotherapy	5 (33.3)	8 (53.3)	13 (43.3)	0.296

*We used two-tailed t-test to compare the differences in TTI and age and Fisher’s exact test to compare the distribution of all other variables.

### Barriers and facilitators

#### Scheduling

Both delayed and non-delayed participants reported that the healthcare team scheduling appointments for them was essential to facilitating treatment initiation. Rather than leaving the burden of scheduling to the patient, setting appointments up immediately after a visit and sending appointment reminders through the MyChart patient portal helped patients keep track of various oncology-related appointments at different times and locations.

62 year-old female, not delayed: “They didn’t rely on me to schedule any appointments. They took care of me. That’s what I mean by they created a treatment plan for me. And I appreciate that. So all I have to do is follow.”

In addition, scheduling was often flexible to accommodate patients’ work schedules and preferences, helping to ensure appointments were accessible and patients could attend them. Patients had varied preferences for appointment timing, with some preferring clustered appointments to reduce travel because they lived far from the hospital, while others found appointments on separate days more convenient to accommodate busy work schedules. This flexibility, in turn, facilitated completing the necessary appointments for clearing, staging, and planning required prior to treatment initiation. However, perspectives on the scheduling system also varied. Although delayed participants found that scheduling itself was easy, next available appointments often required long wait times, while non-delayed participants reported that tests were scheduled quickly:

62 year-old female, delayed: “Not every appointment is easy to get obviously but the center is usually open 7 a.m. to 7 p.m. [and] now they’re offering Saturday appointments like MRIs. Of course sometimes the waiting time is a bit long to get an appointment but I found that given that my diagnosis perhaps … did not delay the MRI.”

63 year-old female, not delayed: “I think they handled that quickly. I think I started, like I said, within, I guess, a comfortable time frame. Because I haven’t had to do, so many, or, you know, various tests. And, they really tried to make sure I was able to be scheduled for all those tests. Like on an emergency basis, because sometimes you have these appointments would take, you know, three months before you get, you know. But they, the staff did a good job of making sure the appointments were scheduled within a given time.”

#### Transportation

Study participants had access to different methods of transportation. Some participants relied on family members to take them to appointments, while others relied on public transportation. Other methods used included ride-shares or cabs paid for by the patient, Access-A-Ride if they qualified, and some participants drove themselves. Most participants (22 of 30 participants) reported having at least partial access to transportation. In addition, proximity of work or home to appointments were reported as crucial for making access to appointments easier.

71 year-old female, not delayed: “Because I had to get people to bring me. My sister. My sister. My aide. Everybody was, hand was on me. So. It was a. I would say it’s an experience of a lifetime.”

While most participants in the study reported having access to transportation, some participants reported transportation issues. These participants were primarily those who had delayed time to treatment initiation. Difficulties with transportation access included relying on public transportation or cabs and having financial difficulties affording those fares. Not being able to afford transportation fares affected patients’ treatment timeliness and contributed to delays. Transportation services that patients qualified for were often unreliable and often showed up late, including hours after the time of their appointment, further contributing to inaccessible appointments and delays in care. Indeed, most non-delayed participants in our study mentioned having transportation access through driving themselves or having family drive them, rather than through public transit, transportation services, or ride-shares; in some cases, this was also a matter of preference due to prior issues with these transportation services:

42 year-old female, delayed: “I was doing all my appointments, screenings on my own. So, car fare and the children and stuff like that, just making sure I can get to where I need to go. There was a few times I felt like, okay, maybe I’ll have to reschedule this. But any rescheduling would push treatment further, and I was afraid of that.”

77 year-old female, delayed: “Sometimes when I had to come over … there wouldn’t be like enough shuttle buses. And I would have to wait and wait. And I was getting more anxious and more anxious, you know.”

62 year-old female, not delayed: “The, in the Medicaid that I have … they provide car service or taxi, something like that. I never, I never use it. Because they never comply. They never, the three times that I call. Never come at the time. And if I call, they call me, they running late … I’m not trying to bother them. Because they never come in a specific time for me to be here early. So that’s the reason why I don’t bother.”

#### Impact on personal finances and financial support

Almost all patients in our study reported receiving adequate financial support to help cover the cost of their care. Patients, regardless of documentation status, obtained health insurance coverage through Medicaid or their employment, limiting out of pocket costs. Additionally, some participants reported obtaining benefits from social security to help cover costs and applied for programs that provided food assistance. This was overall consistent across delayed and non-delayed participants.

However, for delayed patients, having monetary assistance through third-parties was not enough to cushion the strain on their finances. Having a cancer diagnosis meant not just needing coverage for treatments, but also paying for transportation to appointments and for childcare during appointments for mothers who needed it. A significant financial challenge among these patients was saving money and preparing to be without an income while receiving treatments. Several patients reported depleting their savings and incurring debt from the financial burdens, and patients were forced to rely on family for financial support.

45 year-old female, delayed: “Yeah, it’s like I have every day, every minute, to hear the phone ringing, and the credit card company calling me nonstop. And I can’t answer it. I don’t know what to say to them. You know?”

50 year-old female, delayed: “I don’t come from, like, a family that has a lot of money … So, I was even, like, I had to just, like, kind of clear it with my siblings … To ask my mom because I would be taking money from, from them, essentially. I just wanted to make sure, like, it was okay with everyone … Which it was. So, my mom helped me out. Like, she gave me a couple thousand dollars every month.”

58 year-old female, delayed: “I stopped shopping … I had to save money because I’ll be out of work for some time.”

#### Time cost

Patients needed to work to support themselves during the time to treatment initiation, and delayed participants especially reported challenges finding time to take off work and attend appointments. Delayed patients stated that long times in waiting rooms, having different medical tests on different campuses, and needing to use personal vacation time to attend appointments all contributed to difficulties with timing. Furthermore, a few young mothers had challenges with figuring out childcare while they were attending appointments, and tending to their children’s needs also hindered making appointments. These factors made it difficult for delayed participants to attend the appointments they needed to complete prior to treatment initiation, and the high number of appointments that patients needed to attend exacerbated these difficulties:

62 year-old female, delayed: “…it was just difficult to get out of work to go to these appointments … you know, requesting early, coming in late, leaving early. Using my personal time for that. My vacation time for that.”

50 year-old female, delayed: “Waiting in the waiting room. You know … When I say really long. I mean more than an hour. Two hours. You know. That’s kind of like. You could plan for an hour. Being an hour late or something. But like. Two hours is getting like ridiculous. So. Yeah. I mean. That part is a little frustrating….”

45 year-old female, delayed: “Like my daughter, I had, it’s just me and her, so it was hard to, you know, taking her with me in some places she cannot enter … I got one and two persons to help, like to stay with her, but not all the time. …People from the church. Because they have jobs, so, you know, I don’t have that luxury all the time … I almost didn’t get to do the stress test … I didn’t have no one else to keep her … Because I have to do all those stuff, and then, as I said, with my daughter, I don’t have help with her.”

### Coping in the interim: waiting for treatment initiation

#### Emotional well-being

Receiving a cancer diagnosis carries a lot of emotions for patients. Participants from both delayed and non-delayed groups reported a vast range of feelings in the time while they waited for treatment, including feeling shocked about their diagnosis, scared of how they would tolerate surgery and chemotherapy treatment, and worried about life and death. Many reported fears and worries tied to financial and logistical concerns, such as the uncertainty of how they would function after treatment and their ability to continue working and supporting themselves or their families. Patients also reported feeling afraid, frightened, lonely, and disconnected from reality. Some patients even started blaming themselves.

50 year-old female, delayed: “…because during those two months, you’re really in limbo. Like, even I remember just walking down the street feeling … very disconnected … A little frustrated with myself. Mostly scared.”

77 year-old female, delayed” “And I said, I can’t believe this. And I was scared. I always presented a strong façade. And I was scared as hell. And I said, God, this is your way of telling me, I’m going to die soon, but I had to collect myself.”

57 year-old female, not delayed: “I was, like, in total shock, you know, because to begin with, there’s no history of breast cancer in my family … And being the head of the family, you know, I was shocked with regards to what’s going to happen with all the people that are depending on me … I was wondering about the mortgage. How am I even going to pay my mortgage? Worry about my immediate family and then the responsibility of just all these people’s life, basically. I didn’t know how I was going to do financially with the medical bills also…”

#### Emotional support

Both delayed and non-delayed patients counted on networks of support to navigate through the period from diagnosis to treatment initiation. Many turned to family and friends for strength and used religion and their faith to cope with uncertainty. Some patients also utilized available counseling services. The period until treatment initiation was stressful for patients, which was difficult to endure without emotional support. These supports not only helped them cope with their diagnosis, but also helped them move through this difficult process and initiate treatment:

77 year-old female, delayed: “So, you know I did my little concentration I did my meditation and finally I said to myself God this is, this is your way of telling me go get it done … so I did it got the biopsy did the whole nine yards, went for the operation, my son and daughter were with me during the operation in the waiting room.”

61 year-old female, delayed: “Yes, I had a lot of support because I had church sisters that actually went through the same thing with, you know, like what I’m going through … They came here, too. She came here … So she encouraged me. She said, just do what you have to do with all your treatments. I was encouraged by them while waiting, while going through the treatments, and while, before waiting. Before starting the treatment, I was encouraged by her. By a church sister.”

55 year-old, not delayed: “But I have my own, I have like my own therapist and they, you know, she helped me. I like, I got Zoom with her every week. Yeah. And she helped me a lot.”

62 year-old, not delayed:*“*Well, my sister … Aside from being traumatized from the whole thing, but she helped me out. You know, she gave me a care package. You know, she brought me a little care package with blankets and lotions. And, you know, stuff that I would need after surgery and all this stuff. And, you know, my husband was there waiting for me after the surgery. My kids were there … They supported me. So I had a lot of support.”

#### Keeping busy and preparations

While patients were waiting for treatment initiation, many reported that focusing on day-to-day responsibilities was an effective distraction while waiting for treatment. Patients kept busy by engaging in their typical activities and not changing their lifestyle in the interim. Among both delayed and non-delayed patients, patients who worked often continued working during this period, and patients who performed domestic labor continued their usual household activities. Some participants also reported spending more time with their family, especially children and grandchildren, in case their time with them was limited.

61 year-old female, not delayed: “I do think that going to work and … living the way that you would on a regular basis … trying to keep everything normal, I think is the way to go. Because if you sit back and really sit down and think about it, you might be overwhelmed … I never asked why me. You know, I never had time to ask why me … So it was a good experience as far as that was concerned.”

85 year-old female, not delayed: “And, you know, nobody wants to hear that. And so, yeah, I was pretty much cool and calm. I did what I had to do. Cooking, cleaning, going to church. Going out to stores with my daughter and my other daughters and my son. And life just went on. I didn’t change anything.”

Keeping busy also included preparing for life during treatment, or sometimes, preparing for their possible mortality. Interestingly, several delayed participants reported making a will and a plan for their family to prepare themselves for what to do if they are no longer around anymore. This finding was not reported in non-delayed participants.

55 year-old female, delayed: “So, yeah. I guess when you’re faced with the fact that the possibility that, you know, something might happen. Yeah. I wanted things in order.”

50 year-old female, delayed: “I made a will … I put all my finances. In my. That might have been something I asked the social worker about … What should I do with all my assets? And stuff like that. Well, I just put everything in my oldest daughter’s name … Like, things I couldn’t just leave open-ended … That was kind of morbid. Like. While I was making lists … Trying to get like. Everything in order. Like. Gave me a little distraction … like I have a 10 year old … you know, you think about all things like that, like, right. I was making lists like to do lists, like God forbid, you know? Cause I have an older daughter, you know? Like this is, you know … These are our to do lists we have to do.”

### Patient perceptions of timeliness

#### Fear of progression

Around half of our cohort, including delayed and not-delayed patients, reported wanting sooner treatment initiation. They described subjectively feeling like they were waiting around for too long for treatment to begin. Often, wanting sooner treatment initiation was due to fears of the tumor growing and progressing while waiting for treatment initiation. In addition, the preference for sooner treatment was also due to alleviating the stress that came with waiting for initiation.

55 year-old female, not delayed: “Yeah, I felt it growing, growing. And then that’s when I told my husband, do I have to wait? That’s, exactly that’s what I thought. I said, if I wait any longer, will it grow any faster?”

62 year-old female, not delayed: “Because you’re not getting no medication. So you’re thinking, you know, I don’t really know the, I’m going to say, the actual time frame of how long does it take for it to, you know, spread to go somewhere else, to metastasize, that’s what you want to say. I don’t know that. But that was scary for me. Okay. Time frame. It should have been a little quicker. I’m sorry. It just should have been a little quicker … I’m home fighting my nails and like, oh my God, is this, you know, so you’re worried. You see, stress alone will kill you. Forget the cancer. Stress alone.”

42 year-old female, delayed: “So, you know, in my mind, this thing is growing. Where’s the chemo? Because as afraid as I am of it, I’m more afraid of cancer. So, to take all these tests first, you know, had me a little worried also because, you know, to me, this mass was not there in my breast. Yes, I found it. But how could it be? You know, it just was not there. Wouldn’t I have found it before? That’s what was going on with me. So you know, the longer I had to wait for the treatment to begin, the more afraid I was, you know. It got worse. I was assuming that it was growing, you know, day by minute…”

#### Preferring time before treatment

Interestingly, some delayed patients reported preferring having more time between treatment initiation and diagnosis. Some intentionally chose a treatment initiation date that afforded them more time to cope and mentally prepare, spend more time with family, put financial and legal affairs in order, and talk to others for second opinions and advice regarding treatment before initiation. From the delayed participant group, patients in approximately equal numbers reported either that they either needed time before treatment or wanted sooner treatment.

55 year-old female, delayed: “I think for me it was the right time. It, you know, helped me just kind of like accept it. Kind of deal with it before I was, you know, rushed into it.”

77 year-old female, delayed: “I knew it was going to take place. And I was preparing myself mentally. For that particular day, you know. And like I said, I kept myself busy with other things whereby I didn’t, you know, get myself crazy over. [It] was good because my mother … My mother died in [that month] … My brother died [that month]. And I did not want to be in that … month. Because I thought that, mentally, I thought that I would be another statistic. And it was a hard … It’s a hard month. And it was a hard thing to do. So I said, make it. The following month. And I preferred [that].”

59 year-old female, delayed: I wouldn’t have changed anything. I wasn’t going to hurry up the surgery. Because I had to find out … I had to find out a second opinion. So I wasn’t going to rush it. Without just having one opinion. I needed time to research and find out. Is this really happening? Is this really what I got? I wouldn’t change anything.”

## Discussion

Our study identified key themes related to time to treatment initiation including barriers and facilitators, emotional coping in the time between diagnosis and treatment, and perceptions of TTI timeliness. Facilitators to treatment initiation included immediate scheduling and appointment reminders, various methods of transportation, and financial support. In contrast, participants whose TTI was delayed reported having extra-clinical barriers to care that were influenced by unmet social needs. These included having challenges with transportation access, financial instability, having to care for others and difficulty receiving time off work to attend medical appointments. Patients across delayed and non-delayed groups described the negative mental and emotional impact of a cancer diagnosis during the time they were waiting for treatment initiation. However, half of the delayed TTI group actually appreciated having the increased time interval before starting treatment, with some choosing to have more time before starting cancer therapy.

Our study found that treatment initiation was facilitated by the healthcare team scheduling appointments quickly and sending patients appointment reminders. Both delayed and non-delayed participants referenced that MyChart specifically helped to facilitate TTI. Interestingly, one study found that MyChart messaging usage among breast cancer patients had differences among different racial and ethnic groups, with non-Hispanic blacks less likely to use electronic messaging (25). Because our study did not systematically assess patients’ preferred communication methods, future research should examine whether communication preferences differ between delayed and not delayed patients and whether this influences TTI. Awareness of these differences may help providers identify barriers patients face in receiving timely care. Discussing with patients their preferred mode of communication, such as MyChart reminders versus telephone calls, may help optimize appointment attendance and prevent treatment delays.

Delayed patients specifically were affected by having appointments across different locations and long waiting times at appointments. These barriers were exacerbated in patients with caregiver responsibilities. A multidisciplinary clinic model or “one-stop” style of cancer care, where cancer patients attend all their appointments in different specialties in one day at the same location, is being explored as model of cancer care for many cancer types at Montefiore Medical Center. A study at Johns Hopkins in stage 2 or 3 breast cancer patients showed that a single day multidisciplinary clinic visit including 2 or 3 specialties significantly reduced the time from cancer diagnosis to first neoadjuvant chemotherapy treatment (26). This model may be especially beneficial for patients who face transportation barriers to attending their appointments. Indeed, one delayed patient in our study, who lived far away from her appointment sites, reported a preference for clustered same-day appointments.

Access to transportation was critical for avoiding delays in treatment initiation. Similar studies have shown that transportation insecurity is a barrier to breast cancer screening and screening mammography completion, with inner city women especially facing transportation barriers when compared with women from a less disadvantaged suburban county (27, 28). Transportation access in our study was affected by distance from home to appointment locations, owning a personal vehicle or having family and friends that could provide transportation rides, as well as dependence on public transportation and car services. In our study, patients who had limited transportation options had to rely on public transit and transportation services such as Access-A-Ride. These transportation modalities were often unreliable, contributing to transportation delays. Counties with high housing and transportation vulnerability have been shown to have higher odds of experiencing surgical delay in breast cancer patients (29). A qualitative study at a healthcare center in Minnesota found that patients receiving cancer treatment reported similar concerns, such as volunteer drivers being unreliable and public transportation being too time intensive (30). Identifying patients who rely on public transportation and car services is critical to preventing delays in their care. Clustering appointments for these patients may be especially helpful for facilitating timely treatment initiation.

Financial toxicity was a significant and multifaceted burden among breast cancer patients at our institution. While most patients were able to receive financial support through insurance, they often required additional assistance to cover various non-medical costs. Indeed, a study at our institution showed that private insurance is associated with decreased risk of delay in treatment initiation (8). Our findings are consistent with Lauver’s theory of care-seeking behavior, which we applied *post hoc* to help interpret our findings. In this framework, socioeconomic factors like health insurance are “facilitating conditions” of healthcare seeking-behavior (31). Delayed patients addressed financial needs by asking for support from family, diving into savings accounts, and even accumulating debt. Delayed patients especially struggled with covering transportation fees, childcare costs, and access to food. Another qualitative study of breast cancer patients found similar sources of short-term financial toxicity–transportation costs, childcare expenses, changing work while receiving treatment–but that the desire to live and get well overshadowed financial costs when making treatment decisions (32). Preparing to have no income while receiving treatments was also a significant source of stress. Preclinical evidence suggests that chronic stress may contribute to tumor progression, as stress-related hormones can influence oncogene expression (33). Clinical studies have also reported associations between psychosocial stress and worse survival outcomes in cancer patients (34). Delays in treatment initiation may prolong the financial toxicity related to unmet social needs, as well as anguish and uncertainty, which may contribute to poor outcomes. Connecting patients with resources not only addresses their unmet social needs but also allows patients to engage meaningfully with the healthcare system and promotes timely treatment.

Approximately half of the total cohort, including delayed and non-delayed participants, expressed that they wished their treatment had been sooner. This suggests that for many patients, their treatment timing was not patient-driven and may reflect system-level barriers. A study comparing cancer care in rural and nonrural areas found that rural patients were more likely to have shorter time to treatment initiation and improved overall survival compared with their urban counterparts (35). This suggests that time to treatment initiation may vary by geographic and healthcare setting, which may demonstrate the influence of system-level barriers on TTI. One study of head and neck cancer patients found that incorporating associate providers–including physician assistants and nurse practitioners–decreased delays in head and neck cancer referral appointments with the surgeon, increasing productivity while maintaining patient satisfaction (36). Incorporating associate providers in breast cancer care may be effective in decreasing TTI. In addition, a study showed that system-wide initiatives that were implemented to minimize time to treatment initiation (reduced median TTI from 43.5 days to 29 days) decreased the average number of visits prior to treatment initiation and lowered overall patients costs (37). More research is needed to identify and implement interventions to reduce TTI and improve patient outcomes.

Many patients experience high levels of stress and fear of disease progression while they wait for treatment initiation. While some participants–both delayed and not delayed–preferred sooner TTI due to their fears, others in the delayed group elected for later treatment dates in order to emotionally prepare for treatment. One study in ovarian cancer patients used shared medical appointments–where a multidisciplinary team of providers provides care to multiple patients at the same time–for health education visits on chemotherapy, and found high patient satisfaction with this model (38). Shared appointments may be a useful and efficient way to improve preparedness and readiness to initiate treatment, while also addressing emotional concerns that may arise.

Among delayed patients, preference for additional time before treatment initiation versus earlier TTI were approximately evenly split. Reasons for preferring later TTI included psychosocial factors such as wanting to spend more time with family, financial and logistical considerations reasons (i.e. “putting affairs in order”), as well was seeking second opinions on treatment options. The salience of this preference within the delayed cohort was a surprising finding. While prior studies on TTI and survival have shown that shorter TTI is associated with lower mortality in cancer patients (2, 3, 39), the current literature does not distinguish between patient-preferred versus system-imposed delay. Future studies should examine if there are differences in the burden of unmet social needs and mortality between these delayed groups. As some patients in our study preferred additional time to address financial and logistical concerns, it is possible that patients who elected for later TTI may have greater unmet needs. Given the challenges experienced while waiting for treatment initiation, providers should explore underlying reasons for preferring later TTI, to connect patients with supportive resources. Connecting these patients to adequate resources for transportation access, financial assistance, and emotional support may facilitate TTI and prevent delays.

Participants in delayed and not-delayed groups had similar distributions in age, race, ethnicity, first treatment modality, and tumor stage. However, since participants were not purposively matched on these characteristics during recruitment, residual differences between groups may have influenced our comparative qualitative analysis. There was a borderline statistically significant result in pathologic diagnoses between the delayed and non-delayed groups, with three delayed participants diagnosed with ductal carcinoma *in situ* (DCIS). Since DCIS is a non-invasive malignancy, differences in treatment timelines compared with invasive breast cancers may have influenced our findings. Although not statistically significant, two delayed participants underwent mastectomies, and the need for more complex surgical planning may have contributed to longer TTIs, potentially confounding comparisons between groups. Finally, our study had a greater proportion of participants who identified as White in the delayed group compared with the non-delayed group (26.7% vs 6.7%), despite well-documented racial disparities in TTI (10). Given our small sample size, this difference may reflect sampling variability, and our study was not powered to draw conclusions regarding racial disparities.

Although qualitative analysis techniques were collaborative and iterative during codebook development, independent coding during the indexing phase may have impacted the findings of this study. Other limitations of our study included interviewing patients at different stages in their treatment course depending on recruitment timing, introducing temporal bias. Although participants were reassured that confidentiality was maintained and that participation would not affect their care, recruitment within the clinical setting and the sensitive nature of these topics may have influenced their responses. Since the number of participants who declined participation was not documented, our ability to assess the presence of non-response bias is limited. Additionally, although participants in our study did not cite the COVID-19 pandemic as a reason for delays in treatment initiation, our study included participants who received care during the pandemic, and it is possible that more delayed patients received care during the pandemic which might influence overall findings. Further, including patients treated with different treatment modalities may have affected the findings of the study. Lastly, although patients were guided during interview to focus on the period between diagnosis and treatment initiation, recall bias may have led to conflation with post-treatment experiences, potentially influencing our findings.

## Conclusion

Access to transportation and financial resources facilitate treatment initiation. Patients who face barriers to transportation access, childcare challenges, and financial toxicity experienced delays in initiating timely treatment. Emotional and logistical support through family, social networks, and religion helped alleviate barriers and cope with emotional stress. Our results highlight a need for more tailored assistance and timely interventions. There is a need to further study how healthcare systems can better support patients in achieving timely treatment initiation.

## Data Availability

The datasets presented in this article are not readily available because interview transcripts and the data generated during this study are confidential to protect patient privacy. Requests to access the datasets should be directed to vikameht@montefiore.org.
