# Supplementary material for: Further assessment of structural and social determinant of health effects on breast cancer care timelines: a qualitative study
Source: Front Oncol. 2026 Aug 19;16:1887906. doi: 10.3389/fonc.2026.1887906 (PMC13533727; doi:10.3389/fonc.2026.1887906)
Supplement: Supplementary file 1 [file DataSheet1.docx]

*Introduction to Study:*

*We would like to tell you more about a study you are eligible for. In this study we want to understand experiences and perspectives of patients diagnosed with breast cancer. We are hoping to identify patterns in care timelines in order to make care more accessible to patients. As part of the study, we will ask you a few questions about the time period between your diagnosis and when you started treatment. We are interested in hearing as much detail as you can provide, and you can stop at any time if you need a break or cannot complete it. Are you willing to participate in this study?*

Patient Cancer History

*I am familiar with some of your history of cancer already. I understand you were diagnosed with breast cancer on _ _ / _ _ / _ _ (MM/DD/YY) and received your first treatment on _ _ / _ _ / _ _ (MM/DD/YY).*

Treatment History

1. I would like to hear about the weeks right before your first treatment.
2. How your life has changed since receiving your cancer diagnosis.
3. I would like to hear more about what made it harder or easier to start treatment for breast cancer. Let’s start with harder. You can begin wherever you like.
   a. If overwhelmingly positive or negative, say: *“Based on all the things you’ve told me, it sounds like you’ve had an overall a positive experience. Now tell me some negative things.”*
4. Can you walk me through the things you needed to do before you could start treatment?
   a. Probe: What were you told you needed to do by your healthcare providers? (Tests, imaging, doctor’s appointments, etc.)
   b. Insurance?
   c. What did you need to do at home? With family? At work etc.?
5. How long did it take before you could start treatment?
   a. Was it sooner or later than you would have liked?
   b. Were there specific insurance issues that you experienced? (Hospital-related…)
6. What were some of your Concerns with starting treatment?
   a. How do you afford getting your healthcare treatments and visits? (Insurance, out of pocket, etc.)

*The rest of this interview we are going to focus on the time period between your diagnosis of breast cancer on [xx date] and the date you began treatment for that cancer. We want to get a big picture of that time period in your life, so we are going to create a timeline of things, people, context that impacted. Specifically, I want to know about what made it easier and harder to eventually begin treatment.*

*Here is a very simple timeline. Where would you like to begin?*

8. How was your treatment explained to you? What was the timeline explained?

Probing on Specific Factors

9. How would you describe your emotional well-being at that time between diagnosis and treatment?
   a. Worry? Stress?
   b. Fear of progression (tumor growing or cancer getting worse)?
   c. Frustration, confusion, anger?
   d. Loneliness?
   e. Anxiety?
   f. Depression?
10. Did you speak to your surgeon/oncologist/health care team about your concerns or your well-being during that time between being diagnosed and starting treatment?
    a. If yes, what did they say?
       i. How was their quality of communication?
       ii. What was your relationship with them like?
    b. If no, why not?

Impression of Delay

11. Was there anything that happened during your experience between receiving your diagnosis and beginning treatment that you think could be improved for future patients?

Conclusion

12. Is there anything else about your care experience, specifically between diagnosis and beginning treatment, that we have not discussed but you would like to tell me more about?
